# Prediction of Suicidality According to Serum Folate Levels in Depressive Patients Receiving Stepwise Pharmacotherapy

**DOI:** 10.3389/fpsyt.2021.747228

**Published:** 2021-12-02

**Authors:** Jae-Min Kim, Ha-Yeon Kim, Hee-Joon Lee, Ju-Wan Kim, Hee-Ju Kang, Sung-Wan Kim, Il-Seon Shin, Byeong Jo Chun, Robert Stewart

**Affiliations:** ^1^Department of Psychiatry, Chonnam National University Medical School, Gwangju, South Korea; ^2^Department of Emergency Medicine, Chonnam National University Medical School, Gwangju, South Korea; ^3^King's College London, Institute of Psychiatry, Psychology and Neuroscience, London, United Kingdom; ^4^South London and Maudsley National Health Service Foundation Trust, London, United Kingdom

**Keywords:** folate, suicidality, prediction, depression, pharmacotherapy

## Abstract

**Background:** The effects of serum folate levels on suicidal behavior, strongly associated with depression, have not been investigated. Therefore, this study investigated the associations between serum folate levels and suicidal behavior in patients with depressive disorders.

**Methods:** Serum folate levels were measured at baseline in 1,094 patients with depressive disorder, 884 of whom were followed during a 12-month period of stepwise pharmacotherapy. Suicidal behaviors evaluated at baseline were (i) previous suicide attempt and (ii) baseline suicidal severity; behaviors evaluated at follow-up were (iii) increased suicidal severity and iv) fatal/non-fatal suicide attempt. Associations of serum folate levels with four types of suicidal behaviors were analyzed using logistic regression models after adjustment for relevant covariates; they were also examined using area under receiver operating characteristic (AUROC) curve analyses.

**Results:** Reduced serum folate levels (<6.0 ng/mL) were independently associated with all four types of suicidal behaviors. AUROC curve analyses indicated that discriminant or prognostic values of reduced serum folate levels were fair for fatal/non-fatal suicide attempt during follow-up, whereas they were modest for previous suicide attempt, baseline suicidal severity, and increased suicidal severity.

**Conclusions:** Serum folate levels could serve as a biomarker of suicidal behavior in depressive patients. However, it should be used as an adjunct rather than a substitute for prediction of suicidal behavior considering its low prognostic values. Further replication studies are needed for its clinical utilization.

## Introduction

Suicide is a major public health problem worldwide. Approximately 800,000 people die by suicide annually, comprising 1.5% of all deaths (1). Non-fatal suicidal behavior, such as suicidal ideation or attempt, is considerably more common than fatal suicide (2). Biomarkers reflecting or predicting suicidal behavior would be helpful for establishment of suicide prevention strategies. Peripheral blood biomarkers for suicidal behavior risk may have several advantages in terms of convenience and cost-effectiveness. However, studies of potential targets (e.g., monoamines, neurotrophins, hypothalamic-pituitary-adrenal axis, and inflammatory markers) have failed to provide consistent results (3, 4).

Folate is involved in one-carbon transfer (methylation) reactions necessary for the production of monoamine neurotransmitters, phospholipids, and nucleotides (5). Folate deficiency, which causes impaired methylation reactions in the central nervous system, has been associated with depressive and neuropsychiatric illnesses (6–8). In contrast, folate intake has been associated with augmentation of antidepressant effects (9, 10). Suicidal behavior is strongly associated with depressive disorder and suicidal ideation is one of the diagnostic criteria for depressive disorders, according to the Diagnostic and Statistical Manual of Mental Disorders, Fifth Edition (11). Folate thus might be associated with suicidal behavior. A previous small study with 18 depressed inpatients reported no difference in serum folate levels between those with and without violent suicide (12). However, to our knowledge, there have been no studies regarding the relationship between folate and various suicidal behaviors in a real world depressive outpatients setting.

Using data from a prospective study of Korean patients with depressive disorders who were receiving stepwise antidepressant treatment strategies, this study investigated the associations of serum folate levels with suicidal behavior in patients with depressive disorders.

## Materials and Methods

### Study Overview

This study was carried out as a component of the MAKE Biomarker discovery for Enhancing anTidepressant Treatment Effect and Response (MAKE BETTER) program. Details of the study have been published as a design paper (13) and the study was registered with cris.nih.go.kr (identifier: KCT0001332). All data concerning sociodemographic and clinical characteristics at baseline, as well as treatment-related variables at follow-up examinations during the acute treatment phase (evaluated at 3, 6, 9, 12 weeks) and the continuation treatment phase (evaluated at 6, 9, and 12 months), were obtained using a structured clinical report form. The data acquisition was performed by clinical research coordinators who were blinded to treatment modalities; these staff were trained in clinical report form implementation and data collection methods by the research psychiatrists. Patient data were recorded on a clinical report form, registered on the website of the MAKE BETTER study (http://icreat.nih.go.kr/icreat/webapps/com/hismainweb/jsp/cdc_n2.live) within 3 days, and monitored by data management center personnel. This study was approved by the Chonnam National University Hospital Institutional Review Board (approval no. CNUH 2012-014).

### Participants

Patients with depressive disorders were consecutively recruited from March 2012 to April 2017 from among patients who had visited the outpatient psychiatric department of Chonnam National University Hospital. All inclusion instances represented new treatment episodes, regardless of whether depressive symptoms were first-onset or recurrent. Inclusion criteria were: (i) age older than 7 years; (ii) diagnosis of major depressive disorder, dysthymic disorder, or depressive disorder not otherwise specified, using the Mini-International Neuropsychiatric Interview (14), a structured diagnostic psychiatric interview based on the Diagnostic and Statistical Manual of Mental Disorders, Fourth Edition (DSM-IV) criteria; (iii) Hamilton Depression Rating Scale (15) score ≥14; (iv) ability to complete questionnaires, understand the objective of the study, and provide written informed consent. Exclusion criteria were as follows: (i) unstable or uncontrolled medical condition; (ii) inability to complete the psychiatric assessment or comply with the medication regimen because of severe physical illness; (iii) current or lifetime DSM-IV diagnosis of bipolar disorder, schizophrenia, schizoaffective disorder, schizophreniform disorder, psychotic disorder not otherwise specified, or other psychotic disorder; (iv) history of organic psychosis, epilepsy, or seizure disorder; (v) history of anticonvulsant treatment; (vi) hospitalization for any psychiatric diagnosis other than depressive disorder (e.g., alcohol/drug dependence); (vii) receipt of electroconvulsive therapy for the current depressive episode; (viii) pregnancy or lactation. All participants reviewed the consent form and provided written informed consent. For participants under 16 years of age, written consent was obtained from a parent or legal guardian, and written assent was obtained from the participant.

### Baseline Characteristics

#### Serum Folate

Participants were instructed to fast (except water) overnight prior to morning blood collection. They were then asked to sit quietly and relax for 25–45 min before blood sample collection. Serum folate levels were measured using an immunoassay kit (Roche) at GreenCross LabCell (Yongin, Korea).

#### Covariates

Sociodemographic characteristics examined in this study were age, sex, year of formal education, marital status (currently married or not), cohabitation status (living alone or not), religion (religious observance or not), occupation (currently employed or not), and monthly income (above or below 2,000 USD). Clinical characteristics evaluated comprised diagnoses of depressive disorders (major depressive disorder or other depressive disorders) with additional specifiers including melancholic and atypical features, age at onset and duration of illnesses, number of previous depressive episodes, duration of present episode, family history of depression, number of concurrent physical disorders (determined using a questionnaire regarding ~15 systems or disorders), smoking status (current smoking or not), and use of vitamin supplementation. Symptom assessment scales were also administered. Depressive and anxiety symptoms were evaluated by the Hospital Anxiety Depression Scale-depression subscale (HADS-D) and anxiety subscale (HADS-A) (16), respectively; screening for alcohol-related problems was performed by using the Alcohol Use Disorders Identification Test (AUDIT) (17). Higher scores were indicative of more severe symptomatology.

### Pharmacotherapy

Details of the treatment in this study have been previously published (13, 18). Before treatment commencement, a comprehensive review was made of each patient's clinical manifestations (e.g., psychotic and anxiety symptoms), severity of illness, physical comorbidities and medication profiles, and history of previous treatments. Minimum and maximum dosages of pharmacological agents were determined in accordance with existing treatment guidelines (19, 20). For initial treatment (Step 1), patients received antidepressant therapy, considering the above data and treatment guidelines (20–22), for 3 weeks. Antidepressants used were bupropion, desvenlafaxine, duloxetine, escitalopram, fluoxetine, mirtazapine, paroxetine, sertraline, venlafaxine, and vortioxetine. After Step 1 antidepressant monotherapy, next-step pharmacotherapy could be administered at 3-week intervals during the acute treatment phase (3, 6, 9, and 12 weeks with a 3-day allowable window) and at 3-month intervals during the continuation treatment phase (6, 9, and 12 months with a 7-day allowable window), as needed. At the end of each pharmacotherapy step, overall effectiveness and tolerability were reviewed to establish measurement-based next-step treatments. In cases of insufficient improvement (Hamilton Rating Scale for Depression score reduction of <30% from baseline) or intolerable side effects, patients were instructed to choose whether they would prefer to remain in the current step or enter next-step strategies with switching (S), augmentation (A), combination (C), S + A, S + C, A + C, and/or S + A + C. When determining treatment strategies, each patient's preference was prioritized to maximize medication compliance and treatment outcomes (23). Antidepressants switched or combined were bupropion, desvenlafaxine, duloxetine, escitalopram, fluoxetine, mirtazapine, paroxetine, sertraline, venlafaxine, and vortioxetine. Augmented drugs were buspirone, lithium, triiodothyronine, and atypical antipsychotics (e.g., aripiprazole, risperidone, olanzapine, quetiapine, and ziprasidone).

### Suicidal Behavior Outcomes

Four types of suicidal behaviors were defined in this study.

(i) Previous suicide attempt: This was defined as the self-reported history of an act of deliberate self-harm at any time before the baseline assessment, accompanied by at least some intent to die, regardless of the objective lethality of the action (24). Ambivalent intent to die at the time of a deliberate self-harm act was included in the definition of a suicide attempt. However, self-injurious behaviors with no suicidal intent or unknown intent were excluded from the definition.
(ii) Baseline suicidal severity: The Brief Psychiatric Rating Scale (BPRS) (25) suicidality item score was used. Participants were asked the following questions: “Have you felt that life wasn't worth living? Have you thought about harming or killing yourself? Have you felt tired of living or as though you would be better off dead? Have you ever felt like ending it all?” If participants reported suicidal ideation, further questions were asked: “How often have you thought about this? Do you have a specific plan?” Participants' responses were recorded as a score of 1–7, then divided into lower [score 1 (not present) to 3 (mild)] and higher [score 4 (moderate) to 7 (extremely severe)] suicidal severity groups.
(iii) Increased suicidal severity: The BPRS suicidality item score was re-evaluated during the 12-month pharmacotherapy period at 3, 6, 9, and 12 weeks, as well as at 6, 9, and 12 months. Any instance of an increase in the score during the follow-up period, compared with the baseline score, was defined as increased suicidal severity.
(iv) Fatal/non-fatal suicide attempt: This was defined as either a suicide attempt, as described above, or death by suicide during the 12-month pharmacotherapy period.

### Statistical Analysis

Sociodemographic and clinical characteristics (e.g., assessment scales at baseline) and treatment steps during the 12-month pharmacotherapy period were compared between higher and lower median serum folate groups using t-tests or χ^2^ tests, as appropriate. These comparisons were repeated according to previous suicide attempt status. Variables associated at conventional levels of statistical significance (*p* < 0.05) in these analyses were regarded as covariates in subsequent adjusted analyses. Serum folate levels were compared among the four suicidal behaviors using the Mann–Whitney *U*-test. Optimal folate level cut-offs for suicidal behaviors were obtained using area under receiver operating characteristic (AUROC) curve analyses. Logistic regression analyses were conducted to investigate the associations of higher and lower optimal folate level cut-offs with suicidal behaviors, considering potential covariates in terms of sociodemographic, clinical, and treatment-related characteristics. Additional analyses were carried out to investigate the effects of folate deficiency (<3 ng/mL) (26) on suicidal behaviors. Statistical analyses were performed using SPSS software, version 25.0.

## Results

### Recruitment

**Figure 1:**
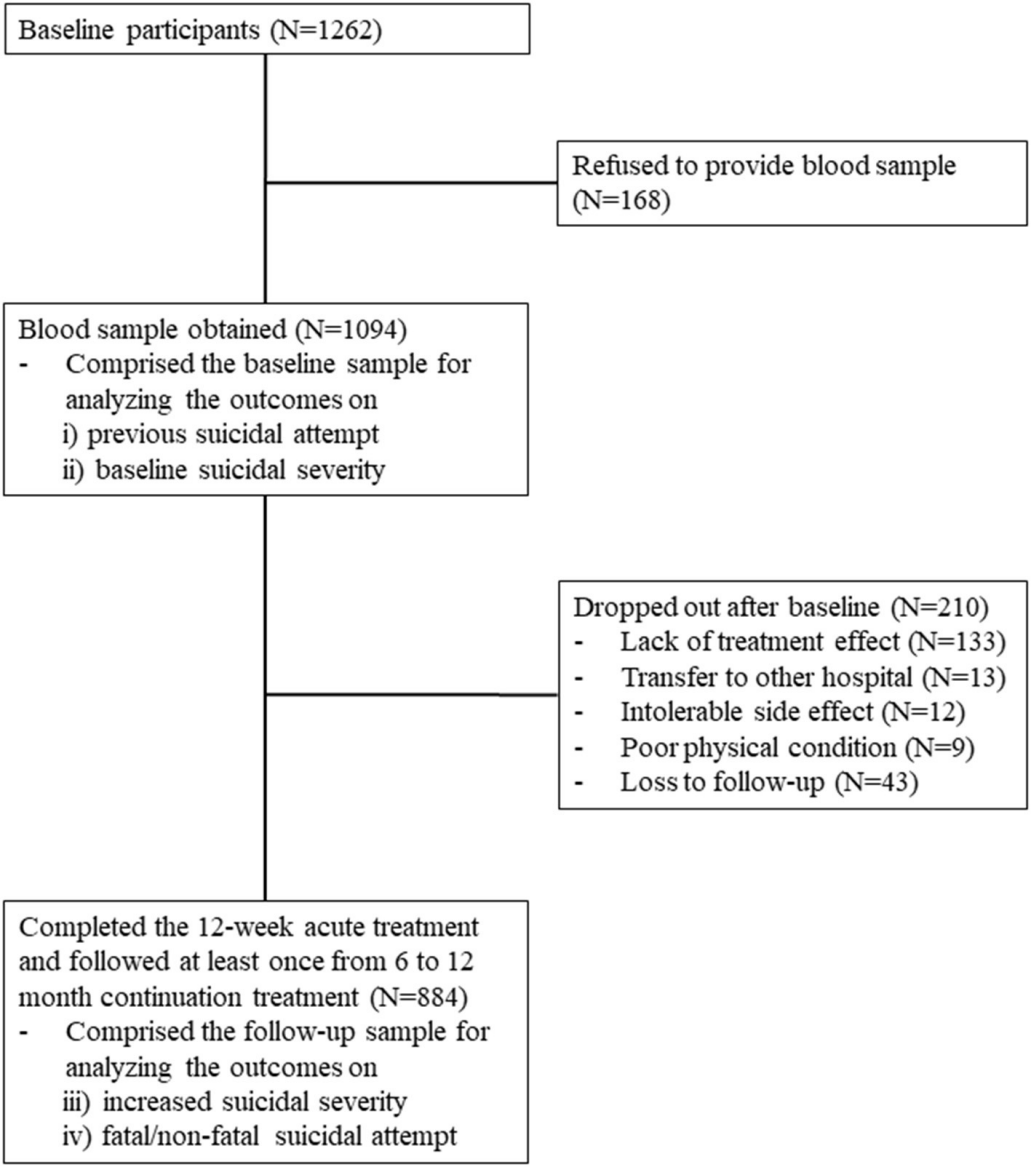
Participant recruitment and attrition.

The recruitment process is summarized in Figure 1. Of 1,262 participants evaluated at baseline, 1,094 (86.7%) provided a blood sample for measurement of serum folate levels and comprised the baseline sample. In this sample, the mean (SD; range) age was 56.9 (14.9; 17 ~ 85) years, 69% (*N* = 753) were female; the mean (SD) duration of education was 9.1 (4.8) years; 30% (*N* = 327) were unmarried; 15% (*N* = 167) were living alone; 29% (*N* = 316) were unemployed; and 60% (*N* = 653) had monthly income <2,000 dollars. Of these 1,094 participants, 884 (80.8%) completed the 12-week acute treatment and were followed up at least once from 6 to 12 months during continuation treatment; these participants comprised the follow-up sample. No significant differences in baseline characteristics were found between participants with or without a blood sample. However, loss to follow-up at 12 months was significantly associated with unemployed status and melancholic features at baseline.

### Characteristics by Serum Folate Levels and Suicidal Behavior

In the baseline sample (*n* = 1,094), median (interquartile range) and mean (standard deviation) values of serum folate levels were 7.4 (6.0) and 8.9 (6.0) ng/mL, respectively. Previous suicide attempt and higher baseline suicidal severity were present in 96 (8.8%) and 362 (33.1%) of the 1,094 baseline participants, respectively. Increased suicidal severity and fatal/non-fatal suicide attempt during the 12-month pharmacotherapy period were present in 155 (17.5%) and 38 (4.3%; 32 non-fatal, 6 fatal) of the 884 follow-up participants, respectively. Table 1 shows characteristics compared according to serum folate above-/below-median value (7.4 ng/mL). Reduced serum folate level was significantly associated with younger age, female sex, unmarried marital status, lower monthly income, lower use of vitamin supplementation, and higher AUDIT scores. Table 2 shows the same characteristics compared according to previous suicide attempt status. A history of a previous suicide attempt was significantly associated with younger age, higher education, unmarried status, no religion, higher monthly income, diagnosis of major depressive disorder, atypical depressive features, earlier age at onset, longer duration of illness, higher number of depressive episodes, higher scores on HADS-A, HADS-D, and AUDIT, and higher number of treatment steps over 12 months. Considering these associations and collinearity among variables, the following covariates were selected for further adjusted analyses: age, sex, marital status, religious affiliation, monthly income, atypical feature, number of depressive episodes, use of vitamin supplementation, and scores on HADS-A and AUDIT for baseline outcomes analyses, plus number of treatment steps for 12-month prospective outcome analyses.

**Table 1:** Characteristics compared by folate levels above/below median value (7.4 ng/mL) at baseline.

	**Higher folate *N* = 546**	**Lower folate *N* = 548**	**Statistical coefficients**	***P*** **-value**
**Socio-Demographic characteristics**				
Age, mean (SD) years	58.0 (12.6)	56.0 (16.8)	*t* = +2.225	0.026
Gender, *N* (%) female	430 (78.8)	323 (58.9)	χ^2^ = 50.043	<0.001
Education, mean (SD) years	9.0 (4.7)	9.2 (4.9)	*t* = −0.970	0.332
Marital status, *N* (%) unmarried	136 (24.9)	191 (34.9)	χ^2^ = 12.909	<0.001
Living alone, *N* (%)	83 (15.2)	84 (15.3)	χ^2^ = 0.003	0.953
Religious non-affiliation, *N* (%)	232 (42.5)	251 (45.8)	χ^2^ = 1.217	0.270
Unemployed status, *N* (%)	146 (26.7)	170 (31.0)	χ^2^ = 2.441	0.118
Monthly income, *N* (%) <2,000 USD	303 (55.5)	350 (63.9)	χ^2^ = 7.971	0.005
**Clinical characteristics**				
Major depressive disorder, *N* (%)	455 (83.3)	478 (87.2)	χ^2^ = 3.302	0.069
Melancholic feature, *N* (%)	87 (15.9)	78 (14.2)	χ^2^ = 0.618	0.432
Atypical feature, *N* (%)	31 (5.7)	38 (6.9)	χ^2^ = 0.731	0.393
Age at onset, mean (SD) years	52.4 (15.0)	51.3 (18.2)	*t* = +1.070	0.285
Duration of illness, mean (SD) years	5.5 (9.8)	4.6 (8.2)	*t* = +1.674	0.094
Number of depressive episodes, mean (SD)	1.1 (1.5)	1.1 (1.5)	*t* = +0.537	0.592
Duration of present episode, mean (SD) months	7.2 (10.3)	7.6 (10.5)	*t* = −0.552	0.581
Family history of depression, *N* (%)	86 (15.8)	74 (13.5)	χ^2^ = 1.106	0.293
Number of physical disorders, mean (SD)	1.6 (1.2)	1.6 (1.3)	*t* = −0.089	0.929
Use vitamin supplement, *N* (%)	30 (5.5)	15 (2.7)	χ^2^ = 5.272	0.022
**Assessment scales, mean (SD) scores**				
Hospital anxiety & depression scale-depression subscale	13.7 (3.9)	13.6 (4.0)	*t* = +0.584	0.559
Hospital anxiety & depression scale-anxiety subscale	11.8 (4.0)	11.8 (4.1)	*t* = −0.250	0.803
Alcohol use disorders identification test	3.9 (7.0)	6.8 (10.2)	*t* = −5.616	<0.001
**Treatment step over 12-month (*****N*** **=** **884)**, ***N*** **(%)**				
Step 1	162 (37.4)	164 (36.4)	χ^2^ = 0.539	0.910
Step 2	141 (32.6)	145 (32.2)		
Step 3	80 (18.5)	29 (20.4)		
Step 4	50 (11.5)	50 (11.1)		

**Table 2:** Characteristics compared by previous suicidal attempt before the study enrolment.

	**Absent *N* = 1,141**	**Present *N* = 121**	**Statistical coefficients**	***P*** **-value**
**Socio-Demographic characteristics**				
Age, mean (SD) years	57.7 (14.7)	47.6 (16.3)	*t* = +6.595	<0.001
Gender, *N* (%) female	801 (70.2)	76 (62.8)	χ^2^ = 2.819	0.093
Education, mean (SD) years	9.0 (4.8)	10.6 (4.5)	*t* = −3.592	<0.001
Marital status, *N* (%) unmarried	335 (29.4)	53 (43.8)	χ^2^ = 10.715	0.001
Living alone, *N* (%)	169 (14.8)	21 (17.4)	χ^2^ = 0.554	0.457
Religious affiliation, *N* (%)	666 (58.4)	46 (37.2)	χ^2^ = 19.950	<0.001
Unemployed status, *N* (%)	328 (28.7)	40 (33.1)	χ^2^ = 0.984	0.321
Monthly income, *N* (%) <2,000 USD	693 (60.7)	57 (47.1)	χ^2^ = 8.428	0.004
**Clinical characteristics**				
Major depressive disorder, *N* (%)	956 (84.7)	113 (93.4)	χ^2^ = 6.719	0.010
Melancholic feature, *N* (%)	180 (15.8)	12 (9.9)	χ^2^ = 2.911	0.088
Atypical feature, *N* (%)	63 (5.5)	20 (16.5)	χ^2^ = 21.573	<0.001
Age at onset, mean (SD) years	53.2 (16.2)	39.4 (17.5)	*t* = +8.859	<0.001
Duration of illness, mean (SD) years	4.5 (8.4)	8.2 (10.8)	*t* = −3.615	<0.001
Number of depressive episodes, mean (SD)	1.0 (1.4)	2.1 (2.1)	*t* = −5.980	<0.001
Duration of present episode, mean (SD) months	7.2 (10.5)	9.2 (10.2)	*t* = −2.007	0.045
Family history of depression, *N* (%)	167 (14.6)	18 (14.9)	χ^2^ = 0.005	0.943
Number of physical disorders, mean (SD)	1.6 (1.3)	1.4 (1.4)	*t* = +1.648	0.100
Use vitamin supplement, *N* (%)	44 (3.9)	3 (2.5)	χ^2^ = 0.578	0.447
**Assessment scales, mean (SD) scores**				
Hospital anxiety & depression scale-depression subscale	13.5 (3.9)	14.3 (4.1)	*t* = −2.008	0.045
Hospital anxiety & depression scale-anxiety subscale	11.6 (4.0)	12.8 (4.2)	*t* = −3.019	0.003
Alcohol use disorders identification test	4.8 (8.2)	10.9 (12.2)	*t* = −5.376	<0.001
**Treatment step over 12-month (*****N*** **=** **884)**, ***N*** **(%)**				
Step 1	470 (41.2)	43 (35.5)	χ^2^ = 9.869	0.020
Step 2	367 (32.2)	32 (26.4)		
Step 3	211 (18.5)	27 (22.3)		
Step 4	93 (8.2)	19 (15.7)		

### Serum Folate Levels by Suicidal Behaviors

**Figure 2:**
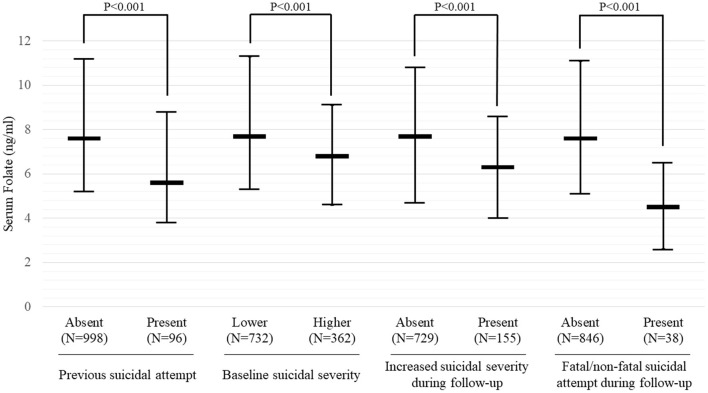
Serum folate levels according to suicidal behavior in patients with depressive disorders. Data are shown as median and interquartile range. Baseline suicidal severity was defined using the brief psychiatric rating scale (BPRS) suicidality item scores, divided into lower [1 (not present) to 3 (mild)] and higher [4 (moderate) to 7 (extremely severe)]. Increased suicidal severity was defined as any instance of an increase in BPRS suicidality item score during the 12-month pharmacotherapy period.

Serum folate levels were compared among the four suicidal behaviors, as shown in Figure 2. Serum folate levels were significantly lower in participants with all four suicidal behaviors than in participants without any suicidal behaviors (all *P* < 0.001). The results of receiver operating characteristic curve analyses for serum folate levels with suicidal behaviors are summarized in Table 3. AUROCs for categorizing the presence of suicidal behaviors were 0.624–0.768 by optimal folate level cut-offs 5.95–6.25 ng/mL; sensitivities were 65.4–80.1%, while specificities were 57.9–74.4%; and positive predictive values were 12.9–43.2%, while negative predictive values were 70.5–97.8%. Folate deficiency (<3 ng/mL) was found in 78 (7.1%) of 1,094 baseline participants and in 65 (7.4%) of 884 follow-up participants. By applying the folate deficiency definition, sensitivities were increased to 94.0–95.8%, but specificities were markedly decreased to 11.2–18.4%; and positive and negative predictive values were similar; 13.8–51.3 and 68.3–96.5%, respectively.

**Table 3:** Results of receiver operating curve (ROC) analyses for serum folate levels with suicidal behavior.

**Suicidal behavior**	**Area under ROC**	**Optimal cut-offs for serum folate levels**	**By optimal cut-offs**				**By folate deficiency (<3 ng/mL)**			
			**Sensitivity %**	**Specificity %**	**Positive predictive value %**	**Negative predictive value %**	**Sensitivity %**	**Specificity %**	**Positive predictive value %**	**Negative predictive value %**
Present previous suicidal attempt	0.684	6.15	70.2	63.3	23.4	93.9	94.8	17.7	20.5	92.1
Higher baseline suicidal severity	0.624	6.25	65.4	59.4	43.2	70.5	95.8	11.2	51.3	68.3
Increased suicidal severity during follow-up	0.649	6.05	67.5	57.9	32.1	85.2	94.0	12.3	26.2	83.2
Fatal/non-fatal suicidal attempt during follow-up	0.768	5.95	80.1	74.4	12.9%	97.8	94.1	18.4	13.8	96.5

*Baseline suicidal severity was defined using the Brief Psychiatric Rating Scale (BPRS) suicidality item scores, divided into lower [1 (not present)~3 (mild)] and higher [4 (moderate)~7 (extremely severe)]. Increased suicidal severity was defined as any instance in the increase in BPRS suicidality item score during 12-month pharmacotherapy*.

### Associations of Serum Folate Levels With Suicidal Behaviors

To maximize both AUROC and sensitivity, a cut-off of 6.0 ng/mL was chosen for the following association analyses. The results of logistic regression analyses regarding the associations of higher (≥6 ng/mL) and lower (<6 ng/mL) serum folate levels with suicidal behaviors are summarized in Table 4. Reduced serum folate levels were significantly associated with all four suicidal behaviors; the strengths of these associations were reduced but remained significant after adjustments for age, sex, marital status, religious affiliation, monthly income, atypical feature, number of depressive episodes, use of vitamin supplementation, scores on HADS-A and AUDIT, and/or treatment step. The results of logistic regression analyses regarding the associations of folate deficiency with suicidal behaviors are summarized in Table 5. Using the cut-off of 6.0 ng/mL, these findings were similar to the above associations with respect to previous suicide attempt, baseline suicidal severity, and fatal/non-fatal suicide attempt; however, the strengths of associations were reduced with respect to increased suicidal severity during the 12-month pharmacotherapy period.

**Table 4:** Associations of serum folate higher (≥6 ng/mL) vs. lower (<6 ng/mL) levels with suicidal behavior in patients with depressive disorder.

**Suicidal behavior**	**Folate level**	***N* patient**	***N* (%) presence of suicidal behavior**	**Odds ratio (95% confidence interval)**	
				**Unadjusted**	**Adjusted^a^**
Present previous suicidal attempt	Higher	688	42 (6.1)	Ref	Ref
	Lower	406	54 (13.3)	2.36 (1.55–3.60)^**‡**^	1.95 (1.21–3.13)^**†**^
Higher baseline suicidal severity	Higher	688	203 (29.5)	Ref	Ref
	Lower	406	159 (39.2)	1.54 (1.19–1.99)^**†**^	1.50 (1.12–2.02)^**†**^
Increased suicidal severity during follow-up	Higher	549	81 (14.8)	Ref	Ref
	Lower	335	74 (22.1)	1.64 (1.16–2.32)^**†**^	1.45 (1.00–2.16)^*^
Fatal/non-fatal suicidal attempt during follow-up	Higher	549	12 (2.2)	Ref	Ref
	Lower	335	26 (7.8)	3.77 (1.87–7.57)^**‡**^	2.69 (1.27–5.69)^*^

*Baseline suicidal severity was defined using the brief psychiatric rating scale (BPRS) suicidality item scores, divided into lower [1 (not present) ~ 3 (mild)] and higher [4 (moderate) ~ 7 (extremely severe)]. Increased suicidal severity was defined as any instance in the increase in BPRS suicidality item score during 12-month pharmacotherapy*.

a *Adjusted for age, sex, marital state, religious affiliation, monthly income, atypical feature, number of previous depressive episode, use vitamin supplement, and scores on Hospital Anxiety and Depression Scale-anxiety subscale and Alcohol Use Disorders Identification Test for baseline analyses, plus treatment steps over 12-month pharmacotherapy for follow-up analyses*.

* *P < 0.05;*

† *P < 0.01;*

‡ *P < 0.001*.

**Table 5:** Associations of folate deficiency (<3 ng/mL) with suicidal behavior in patients with depressive disorder.

**Suicidal behavior**	**Folate level**	***N* patient**	***N* (%) presence of suicidal behavior**	**Odds ratio (95% confidence interval)**	
				**Unadjusted**	**Adjusted^a^**
Present previous suicidal attempt	Normal range	1,016	80 (7.9)	Ref	Ref
	Deficiency	78	16 (20.5)	3.02 (1.67–5.48)^**‡**^	2.32 (1.18–4.49)^*^
Higher baseline suicidal severity	Normal range	1,016	322 (31.7)	Ref	Ref
	Deficiency	78	40 (51.3)	2.27 (1.43–3.61)^**‡**^	2.23 (1.34–3.69)^**†**^
Increased suicidal severity during follow-up	Normal range	819	138 (16.8)	Ref	Ref
	Deficiency	65	17 (26.2)	1.75 (0.98–3.13)	1.42 (0.77–2.64)
Fatal/non-fatal suicidal attempt during follow-up	Normal range	819	29 (3.5)	Ref	Ref
	Deficiency	65	9 (13.8)	4.38 (1.98–9.70)^**‡**^	2.84 (1.19–6.77)^*^

*Baseline suicidal severity was defined using the brief psychiatric rating scale (BPRS) suicidality item scores, divided into lower [1 (not present) ~ 3 (mild)] and higher [4 (moderate) ~ 7 (extremely severe)]. Increased suicidal severity was defined as any instance in the increase in BPRS suicidality item score during 12-month pharmacotherapy*.

a *Adjusted for age, sex, marital state, religious affiliation, monthly income, atypical feature, number of previous depressive episode, use vitamin supplement, and scores on Hospital Anxiety and Depression Scale-anxiety subscale and Alcohol Use Disorders Identification Test for baseline analyses, plus treatment steps over 12-month pharmacotherapy for follow-up analyses*.

* *P < 0.05;*

† *P < 0.01;*

‡ *P < 0.001*.

## Discussion

In this study of depressive patients receiving a stepwise pharmacotherapy intervention in a real-world clinical setting, reduced serum folate levels were significantly associated with previous suicide attempt history, baseline suicidal severity, and prospective worsening of suicidality and actual suicide attempt or death during the 12-month follow-up period. These findings remained statistically significant following adjustment for multiple relevant covariates.

There are several possible explanations for these findings. First, folate is a B vitamin and an important dietary nutrient. There is increasing observational evidence that nutritional deficits such as low body weight (26) and low levels of cholesterol (27) and docosahexaenoic acid (28) are associated with suicidal behavior. Second, nutritional deficits (i.e., fatty acid deficiency) had negative effects on function with respect to neurotransmitters, the hypothalamic-pituitary-adrenal axis, and limbic and cortical brain areas (29, 30). Folate deficiency also caused impaired methylation reactions and thus impaired the production of neurotransmitters, phospholipids, and nucleotides (5). This dysfunction may lead to suicidal behavior, rather than healthy adaptive behavior, under stressful situations. Third, nutritional deficits such as folate deficiency have been associated with risks of depressive disorders (6, 8, 31), and nutritional intake (including folate intake) has augmented antidepressant effects (9, 32). Suicidal behavior could have similar associations because it is strongly related to depressive disorders (11).

Reduced serum folate level (<6.0 ng/mL) and folate deficiency (<3.0 ng/mL) were both significantly associated with suicidal behavior. These findings are consistent with previous observations concerning associations of reduced serum folate levels (but not folate deficiency) with depressive disorders (6, 33). Similarly, low and high levels of docosahexaenoic acid (stratified according to the median value) influenced suicide risk (28). With respect to the strengths of the associations, lower serum folate levels were significantly associated with all four suicidal behaviors evaluated in this study, while folate deficiency was not associated with increased suicidal severity during the 12-month pharmacotherapy period. These results were presumably influenced by the low statistical power related to the low number of participants in the folate deficiency group (*N* = 65) compared to the lower serum folate levels group (*N* = 335) (see Tables 4, 5). The low prevalence of folate deficiency is likely to be due to the high intake of folate-containing green vegetables in Korean populations, compared with Caucasian populations (34).

According to AUROC curve analyses, discriminant or prognostic values of reduced serum folate levels (<6.0 ng/mL) were fair for fatal/non-fatal suicide attempts during follow-up, but modest for previous suicide attempt, baseline suicidal severity, and increased suicidal severity (Table 3). This stronger association with actual prospective suicide attempts was consistent with previous findings concerning other nutrients: low cholesterol was associated with violent (rather than non-violent) suicide attempt (35), while lower docosahexaenoic acid levels were predictive of suicide attempt over a 2-year period (28). The associations of reduced serum folate levels with previous suicide attempt and baseline suicidal severity might be obscured by selection bias because people with both nutritional deficiency and higher suicidality are more likely to be hospitalized (or die of suicide) and therefore under-represented in an outpatient treatment setting (36). When applying the folate deficiency criteria, the biomarker values became unbalanced with respect to sensitivities and specificities. Based on these observations, lower folate levels (rather than folate deficiency) could be recommended to distinguish between previous suicide attempt or present suicidal severity, as well as for prediction of suicidal behaviors during the treatment period. With respect to predictive values, positive predictive values were obvious low, while negative predictive values were considerably high. The low positive predictive values probably due to the low prevalence or incidence of suicidal behavior, particularly in actual retrospective and prospective suicidal attempt. On the other hand, the high negative predictive values might be related to higher sensitivity than specificity (37). Summing up, the serum folate level could be used as an adjunct rather than a substitute for prediction of suicidal behavior considering its low positive predictive values.

### Limitations and Strengths

This study had several limitations. First, baseline serum folate levels were measured at only one time point; therefore, it was not possible to assess the association between potential changes in folate levels and prospective suicidal behavior. Second, the effects of earlier depressive symptoms on oral intake were not evaluated, although depression severity did not differ according to folate level in this cohort. Third, because of the observational study design, treatment was decided by patient preference with a physician's guidance, rather than using a pre-established protocol; thus, inter-physician variability might have affected the outcomes. Fourth, there was considerable sample attrition during the 12-month treatment period. Because of poor prognostic characteristics among participants who were lost to follow-up, such as unemployed status and melancholic features, these participants presumably would have attenuated (rather than exaggerated) the observed findings. However, serum folate levels did not differ according to follow-up status. Fifth, this study was carried out at a single center, which might have limited the generalizability of the findings. Sixth, the number of fatal suicide during the 12-month pharmacotherapy was too small to analyze separately.

This study had multiple strengths, including its novel combined retrospective and prospective design for evaluation of suicidal behaviors. The sample size was large; participants were evaluated with a structured research protocol, as well as well-recognized and standardized scales. Diverse covariates (e.g., vitamin supplementation status) were considered, which could have influenced the study findings.

## Conclusion

Our findings indicate that serum folate level could serve as a biomarker for identification and prediction of suicidal behavior before and after pharmacological treatment in patients with depressive disorders. The observed suicide rate during the 12-month follow-up period was 678 per 100,000 (6/884), ~27-fold higher than the observed suicide rate (24.6 per 100,000) among all South Koreans in 2019 (38). For clinical application, considering the low specificity and positive predictive value, further studies are needed to replicate this finding in other populations and to investigate the effects of folate intake on suicidal behavior prevention in randomized controlled trials.

## Data Availability Statement

The raw data supporting the conclusions of this article will be made available by the authors, without undue reservation.

## Ethics Statement

The studies involving human participants were reviewed and approved by Chonnam National University Hospital Institutional Review Board. Written informed consent to participate in this study was provided by the participants' legal guardian/next of kin.

## Author Contributions

J-MK had full access to all of the data in the study and take responsibility for the integrity of the data and the accuracy of the data analysis and obtained funding. H-JK and J-MK contributed to the study concept and design. H-YK, H-JL, and BC contributed to data acquisition. S-WK, RS, BC, and J-MK conducted to analyze and interpret the data. J-WK, RS, S-WK, and J-MK wrote the drafting of the manuscript. J-WK, RS, H-JK, S-WK, and J-MK contributed to critical revision of the manuscript for important intellectual content. RS, S-WK, and J-MK performed statistical analysis. Additionally, H-JL, RS, S-WK, and J-MK contributed to administrative, technical, or material support. S-WK and J-MK supervised the overall study process. All authors contributed to the article and approved the submitted version.

## Funding

The study was funded by grants of National Research Foundation of Korea Grant (NRF-2019M3C7A1031345 and NRF-2020M3E5D9080733). RS was part-funded by the National Institute for Health Research (NIHR) Biomedical Research Center at South London and Maudsley NHS Foundation Trust and King's College London, and from the National Institute for Health Research (NIHR) Applied Research Collaboration South London (NIHR ARC South London) at King's College Hospital NHS Foundation Trust. RS was also a National Institute for Health Research (NIHR) Senior Investigator. The funders had no role in study design; in the collection, analysis, and interpretation of data; in the writing of the report; or in the decision to submit the paper for publication.

## Conflict of Interest

RS declares research support received within the last 3 years from Janssen, GSK, and Takeda. The remaining authors declare that the research was conducted in the absence of any commercial or financial relationships that could be construed as a potential conflict of interest.

## Publisher's Note

All claims expressed in this article are solely those of the authors and do not necessarily represent those of their affiliated organizations, or those of the publisher, the editors and the reviewers. Any product that may be evaluated in this article, or claim that may be made by its manufacturer, is not guaranteed or endorsed by the publisher.
